# Pregnancy-Induced Hypertension Pathophysiology and Contemporary Management Strategies: A Narrative Review

**DOI:** 10.7759/cureus.63961

**Published:** 2024-07-06

**Authors:** Garima S Agarwal, Anil K Agrawal, Daksh Singhal, Dushyant Bawiskar, Saylee S Shedge

**Affiliations:** 1 Pathology, Jawaharlal Nehru Medical College, Datta Meghe Institute of Higher Education and Research, Wardha, IND; 2 Psychiatry, Raja Rajeshwari Medical College, Bangalore, IND; 3 Sports Physiotherapy, Abhinav Bindra Targeting Performance, Bangalore, IND; 4 Sports Physiotherapy, Ravi Nair Physiotherapy College, Datta Meghe Institute of Higher Education and Research, Wardha, IND

**Keywords:** afi, fetal monitoring, placental dysfunction, preeclampsia, eclampsia, pih

## Abstract

In the case of PIH, the history is the story of gradually developing awareness and the gradual formation of requisite knowledge. The development of the sphygmomanometer, or blood pressure cuff, in the late 1700s, provided the basis for modern systematic blood pressure reporting for Gravid patients. In the following years and over a few decades, the relationship between high blood pressure and these complications, such as preeclampsia and eclampsia, became clearer. The hypertensive disease was categorized by the American Committee on Maternal Welfare in 1952, which included PIH, chronic hypertension, and preeclampsia. Today, attention is being paid to the identification of such factors, the search for ways to enhance the treatment of diseases, methods for their diagnosis, and the enhancement of pregnancy outcomes. Pregnancy can cause high blood pressure in two of the following ways: preeclampsia and gestational hypertension. These conditions are both part of something called pregnancy-induced hypertension (PIH). In the world, most problems for moms and babies during pregnancy come from PIH. To help both mom and baby, we need to know a lot about what causes it, how to manage it, and how to watch the baby carefully. Aspects like immune responses, the environment, and genes all mix to cause PIH. They make the placenta not work right. When the cells that help the placenta grow don't do their job well, when blood vessels are stiff, when there's too much stress on the body, or when there's not a good balance of chemicals that help build blood vessels, things can get bad. Blood vessels all over the body squeeze tight, blood flow goes down, and blood pressure goes up. That can make a lot of organs stop working right and stop the baby from healthy growth. Various studies concluded that PIH severely limits the blood flow to the placenta and thus contributes to reduced fetal growth. It showed that compared to other hospitals, women who experience PIH are more likely to give birth early before the baby is ready, that is, before 37 weeks, and may cause further health complications to the baby. This normally makes the offspring have low birth weight and exposes them to many complications in infancy and the future in case they are born to mothers with PIH. In severe cases, PIH may lead to the death of the infant either by stillbirth or immediately after birth. The researchers have noted several predisposing factors to PIH, which include histories of elevated blood pressure, diabetes, being overweight or obese, and having a family history of PIH. Educating women about the presence of PIH and its causes can help them consult health facilities early, thus helping leaders in achieving better pregnancy results.

## Introduction and background

Pregnancy-induced hypertension (PIH) is a commonly occurring gestational issue that can complicate around a tenth of the total gravid cases and is among the foremost causes of mortality in technologically advanced parts of the world. One of the reasons behind increasing instances of PIH among gravid women is due to underlying cardiometabolic diseases. These diseases have a substantial impact on pregnant women's health and can make a difference through morbidity and mortality outcomes [1]. A variety of disorders affecting the cardiovascular and metabolic systems are included in the category of cardiometabolic disease in fertile women. There is a growing recognition that these disorders play a major role in the morbidity and mortality of this population. Risk factors include obesity, insulin resistance, dyslipidemia, polycystic ovarian syndrome (PCOS), lifestyle, and genetic predisposition. These risk factors should be monitored for early detection of PIH. As metabolic demand increases during pregnancy, disorders are predisposed to occur among a large section of cases. Throughout pregnancy, the mother's circulatory system undergoes significant modifications to ensure the placenta gets enough blood and to meet the growing fetus's increased metabolic needs [2]. Enhanced cardiac output, elevated plasma volume, and decreased systemic vascular resistance are some of these hemodynamic alterations. Hemodynamic adaptations in gravid situations include increased cardiac output and plasma volume, along with a reduction in systemic vascular resistance.

When high blood pressure occurs during pregnancy, after 20 weeks of gestation, without proteinuria or other preeclampsia symptoms, the condition is known as gestational hypertension. If left untreated, it may develop into more serious disorders such as persistent hypertension or preeclampsia. The physiological stress arising due to the gravid situation can take a toll on the mother as well as the fetus in the form of morbidity and mortality. Adapting to the physiological changes, trimester-wise analysis shows that PIH in the first trimester is rare; if occurring, it might be the result of underlying morbidity or hypertension condition [3]. Renal disease, endocrine abnormalities, and cardiovascular issues are examples of potential secondary causes. Thorough analysis to separate secondary causes of hypertension from chronic hypertension. Pregnancy-safe antihypertensive drugs such as nifedipine, labetalol, or methyldopa may be started or stopped. Frequent prenatal checkups include monitoring blood pressure and looking for end-organ damage indicators. After 20 weeks of pregnancy, usually in the second trimester, PIH commonly occurs among the concerned population. It can be considered a precursor to preeclampsia. Regular blood pressure checks and urine tests for proteinuria are advised in addition to dietary, exercise, and salt reduction modifications for improved management in order to detect proteinuria early. The most crucial time for the development of preeclampsia and gestational hypertension, as well as PIH, is during the third trimester. Greater than 140/90 mm Hg in blood pressure, proteinuria can be present or not. Possibility of developing into severe eclampsia or preeclampsia. There is an elevated danger of problems for both the mother and the fetus in the third trimester. Pregnancy-induced hypertension is managed differently in each trimester due to the condition's changing nature and possible course. To reduce risks and guarantee positive outcomes for the mother and the unborn child, early detection, suitable interventions, and close monitoring are crucial throughout the pregnancy [4].

## Review

Pregnancy-induced hypertension pathophysiology and contemporary management strategies

Table 1 depicts a summary of articles included in the review.

**Table 1:** Summary of articles included in this review PIH: Pregnancy-induced hypertension, HDP: Hypertensive disorders in pregnancy, IUGR: Intrauterine growth retardation

Author	Year	Type	Conclusion
Khedagi et al. [5]	2021	Original article	The prevalence of hypertensive diseases is rising, and they are a major source of illness and mortality among mothers and fetuses. Furthermore, there are racial/ethnic differences in the prevalence, morbidity, and mortality rates linked to hypertension diseases.
Garovic et al. [6]	2022	article	Pregnancy antihypertensive medication The incidence of severe hypertension is halved by any form of hypertension. This is convincing enough to some, if not most, to force a shift in practice in the direction of more harsh therapy.
Metoki et al. [7]	2022	Review article	Measuring blood pressure during pregnancy is essential for identifying pregnancy-induced hypertension. Determining the prognosis and clarifying the pathophysiology of pregnancy-induced hypertension will continue to require accurate measurement and assessment of blood pressure, including its fluctuation.
Fururya et al. [8]	2008	article	It is vital to look into rodent PIH models as well as human instances in order to gain better knowledge and management of PIH. In these models, the mechanism of placental malfunction and IUGR under maternal hypertension may be closely observed.
Kintiraki et al. [9]	2014	article	In summary, PIH is a prevalent health issue that has negative consequences for the mother as well as the fetus or newborn. It is thought to be a complex medical illness for which the pathogenetic process is still being worked out. Further research elucidating the latter will also help improve medical care and optimize the outcome of pregnancy.
Sinkey et al. [10]	2021	Original article	The best ways to lower the morbidity and death rate from HDP are to follow a policy that encourages early identification and treatment, as well as to test for blood pressure during prenatal and postpartum visits universally.
Magnus et al. [11]	2008	Original article	The findings imply that engaging in recreational physical exercise while pregnant lowers the chance of developing preeclampsia. There was a 20% lower risk for the women who reported engaging in regular physical activity.
Gurjar et al. [12]	2019	Original article	Compared to methyldopa, labetalol lowers blood pressure more quickly and efficiently at both the systolic and diastolic levels. Methyldopa and labetalol have comparable side effects and safety profiles.
Opichka et al. [13]	2021	Article	However, it is known that vascular abnormalities occur during all phases of preeclampsia, starting during pregnancy and continuing long after delivery.

Pre-eclampsia

Preeclampsia is a multisystem illness unique to pregnancy that typically manifests after 20 weeks of gestation. In addition to affecting the liver, kidneys, brain, and blood clotting mechanisms, it is typified by newly developed hypertension and proteinuria. There are serious hazards to the mother and the fetus when preeclampsia worsens and develops into eclampsia, which includes seizures. Those altered spiral arteries are not remodeled, and the placental perfusion and the degree of ischemia are reduced. Soluble fms-like tyrosine kinase-1 and soluble endoglin anti-angiogenic factors are increased in the maternal plasma due to placental hypoxia. These elements lead to extensive endothelial dysfunction, which exacerbates organ damage and hypertension - a modified mother's immune system reaction to placental paternal antigens. Preeclampsia risk is increased by specific genetic predispositions and family history [14].

Placental dysfunction

The pathogenesis of PIH, which includes diseases like pre-eclampsia and gestational hypertension, is mostly influenced by placental malfunction. Abnormal growth and function of the placenta can set off a series of events that might result in systemic problems, including hypertension in the mother. The placental cells known as trophoblasts invade the mother's spiral arteries in the early stages of pregnancy, converting them into high-capacity vessels that can supply the placenta with more blood. This invasion frequently occurs in PIH, although it is superficial and partial, which results in insufficient spiral artery remodeling. Placental perfusion is decreased because the spiral arteries continue to be high-resistance and thin. Pro-angiogenic factors (vascular endothelial growth factor, placental growth factor) and anti-angiogenic factors (soluble fms-like tyrosine kinase-1, soluble endoglin) must be in balance for normal placental development [15].

Pro-angiogenic factors are somewhat lacking in PIH, while anti-angiogenic factors are overproduced. Reduced ability to build new blood vessels, which further reduces placental blood flow and the exchange of nutrients. Oxidative stress is caused by ischemia, or a decreased oxygen supply, which results from inadequate blood flow to the placenta. In addition to harming placental cells, oxidative stress causes the mother's blood to become contaminated with anti-angiogenic substances and inflammatory cytokines. Disruption of the systemic endothelium raises the risk of maternal hypertension and other issues. Oxidative stress is caused by ischemia, or a decreased oxygen supply, which results from inadequate blood flow to the placenta. In addition to harming placental cells, oxidative stress causes the mother's blood to become contaminated with anti-angiogenic substances and inflammatory cytokines. Disruption of the systemic endothelium raises the risk of maternal hypertension and other issues. Systemic endothelial dysfunction, proteinuria, and other issues are consequences of placental malfunction [16].

Endothelial dysfunction

A key component of the pathogenesis of PIH, which includes diseases like preeclampsia and gestational hypertension, is endothelial dysfunction. A vital function of endothelium, the thin layer of cells lining blood arteries, is to preserve vascular homeostasis and health. When these cells malfunction, a series of vascular anomalies are set off, which in turn contribute to hypertension and other systemic problems during pregnancy. Blood vessel development and endothelial health are promoted by vascular endothelial growth factor (VEGF) and placental growth factor (PlGF). Angiogenesis is inhibited by soluble endoglin (sEng) and soluble fms-like tyrosine kinase-1 (sFlt-1). Pro-angiogenic factors are decreased, and anti-angiogenic factors are overproduced in PIH. Reactive oxygen species (ROS) are produced when ischemia-reperfusion damage results from inadequate placental perfusion. Endothelial cells are harmed by ROS, which lowers the availability of nitric oxide (NO) and encourages vasoconstriction. Tumor necrosis factor-alpha (TNF-α) and interleukin-6 (IL-6) are two inflammatory cytokines that are released in response to placental ischemia. These cytokines cause both activation and malfunction of endothelial cells. Systemic inflammation is exacerbated by this, leading to increased vascular permeability and leukocyte adhesion [17].

Immune maladaptation

The pathophysiology of pregnancy-induced hypertension (PIH), which includes gestational hypertension and preeclampsia, heavily involves immune maladaptation. A delicate balance between immunity to pathogens and tolerance to the semi-allogenic fetus characterizes the immune system's function in a typical pregnancy. This equilibrium is upset in PIH, which results in aberrant immunological reactions that exacerbate hypertension and endothelial dysfunction. The immune system of the mother is an important regulator of trophoblast invasion. Insufficient immunological tolerance in PIH may result in superficial trophoblast invasion. This causes poor spiral artery remodeling, which lowers placental perfusion and causes ischemia. In PIH, there is a decrease in anti-inflammatory cytokines (such as IL-10) and an increase in pro-inflammatory cytokines (like TNF-α and IL-6). Immune maladaptation's effects include the endothelium being harmed by oxidative stress and chronic inflammation, which decreases its capacity to produce nitric oxide (NO) and other vasodilators. Vascular permeability and extensive endothelial activation are caused by persistent inflammation. Fetal growth restriction and an increased risk of unfavorable pregnancy outcomes result from reduced placental perfusion. A major contributing element to the development of pregnancy-induced hypertension is immune maladaptation. Endothelial dysfunction and systemic inflammation are the results of a complex interplay between immune cell malfunction, cytokine imbalance, and the overproduction of anti-angiogenic molecules. A major contributing element to the development of pregnancy-induced hypertension is immune maladaptation. Endothelial dysfunction and systemic inflammation are the results of a complex interplay between immune cell malfunction, cytokine imbalance, and the overproduction of anti-angiogenic molecules [18].

Genetic and epigenetic factors

PIH, which includes disorders including gestational hypertension and preeclampsia, is mostly influenced by genetic and epigenetic factors. Comprehending these variables aids in the identification of possible biomarkers and treatment targets and offers insights into the intricate pathophysiology of PIH. PIH, especially preeclampsia, frequently occurs in families, indicating a possible genetic inclination. Women who have a mother or sisters who have had Parkinson's disease are more vulnerable. Angiotensin (AGT) gene variations are associated with a higher incidence of preeclampsia and hypertension. Variations in this gene are linked to blood pressure regulation and vascular resistance. Changes may impact the synthesis of nitric oxide, which may impact endothelial function and vascular tone. Elevated homocysteine levels have been associated with certain polymorphisms that may compromise endothelial function. Preeclampsia rates are higher in pregnancies fathered by males who were born from preeclamptic pregnancies, suggesting that genetic contributions from the father can also impact the risk of PIH.

Vascular reactivity and blood pressure regulation

Vascular reactivity and blood pressure regulation are significantly altered in PIH, which includes diseases such as gestational hypertension and preeclampsia [19]. These changes play a role in the emergence of hypertension and its related consequences. Strong vasodilator NO, which is normally produced by the endothelium, aids in maintaining vascular tone. Vasoconstriction results from endothelial dysfunction in PIH, which lowers NO production. Elevated levels of endothelin-1 (ET-1), a powerful vasoconstrictor, are linked to hypertension and increased vascular tone in PIH. Reactive oxygen species (ROS) production that is elevated in PIH results in oxidative stress, which destroys endothelium and further lowers NO availability. ROS can increase reactivity to vasoconstrictors by directly impairing the function of vascular smooth muscle. In PIH, elevated levels of inflammatory cytokines, such as TNF-α and IL-6, are observed. RAAS activity is adjusted during a typical pregnancy to account for an increase in plasma volume. Angiotensin II is a strong vasoconstrictor, and aldosterone increases salt and water retention in PIH due to dysregulation of RAAS. Elevated blood pressure is a result of enhanced cardiac output and vasoconstriction caused by increased SNS activity in PIH [20].

SNS activity can be further stimulated by mediators of inflammation and stress. Vasoconstrictors such as angiotensin II and norepinephrine cause the vascular smooth muscle cells in PIH to become more responsive. Vasoconstriction is made worse by reduced sensitivity to vasodilators (such as NO and prostacyclin). Baroreceptors, which sense variations in artery wall stretch to help control blood pressure, may become less sensitive in peripheral ischemic heart disease. This deficit results in a diminished capacity to counteract hypertension [21]. Preeclampsia has traditionally been treated mainly symptomatically, with an emphasis on neuroprotection, maintaining appropriate blood pressure ranges, and prophylactic seizure treatment. Severe cases have been treated with rapid delivery at term or 34 weeks. However, vascular abnormalities have been shown to occur at every stage of preeclampsia, starting with placentation and continuing long after delivery [22]. These abnormalities are most likely the result of a confluence of factors, including inadequate trophoblast invasion, inadequate placental oxygen extraction, a pro-inflammatory immune environment, antiangiogenic factors, endothelial dysfunction, and oxidative stress [13].

Renal association

A crucial component of the pathogenesis and clinical presentation of PIH, which includes diseases such as gestational hypertension and preeclampsia, is renal involvement. Blood pressure regulation is greatly influenced by the kidneys, and malfunction in these organs can lead to the onset and advancement of hypertension during pregnancy. The etiology, clinical consequences, and therapy of renal involvement in PIH are examined here. In PIH, the glomerular endothelium of the kidneys is especially susceptible to injury [23]. Enhanced permeability and reduced vasodilation are the results of endothelial dysfunction. As a result of protein leakage across the compromised glomerular filtration barrier, proteinuria-a defining feature of preeclampsia-occurs. Preeclampsia is frequently associated with this particular condition, which is characterized by glomerular endothelial cell enlargement and capillary lumen blockage. Angiotensin II and aldosterone levels rise as a result of RAAS dysregulation in PIH, which encourages salt and water retention and raises blood pressure. Renal vascular injury and inflammation are associated with elevated levels of ROS and inflammatory cytokines, such as TNF-α and IL-6. The morbidity linked to pregnancy-induced hypertension is mostly influenced by renal involvement in the illness. The pathways encompass oxidative stress, RAAS activation, glomerular endotheliosis, and endothelial dysfunction. For the purpose of identifying and treating PIH, clinical signs such as proteinuria, increased serum creatinine, and oliguria are essential.

Pre-existing renal illness and hypertension both raise the risk of unfavorable pregnancy outcomes. This is especially true for superimposed pre-eclampsia, which can lead to premature delivery, fetal growth restriction, and worsening renal function [24]. Pregnant women with hypertension problems were more likely to develop end-stage renal disease. Women with preeclampsia or eclampsia were at significantly higher risk than those with pregnancy hypertension alone [25].

Management of pregnancy-induced hypertension

PIH is managed with a multimodal approach that focuses on blood pressure control, fetal and maternal well-being monitoring, and safe delivery preparation. This includes gestational hypertension and preeclampsia. The objectives are to treat any complications that develop, reduce the risks to the mother and child, and stop the condition from getting worse and leading to severe preeclampsia or eclampsia. By employing certain strategies, namely, regular monitoring, lifestyle modifications, pharmacological management, delivery planning, postpartum care, and fetal monitoring, we can manage the issue of pregnancy-induced hypertension [26]. Adjusting one's lifestyle is essential for treating PIH, which includes preeclampsia and gestational hypertension. These changes are intended to lower blood pressure, enhance general health, and lower the likelihood of problems for both the mother and the unborn child. Stress the importance of eating a diet high in fruits, vegetables, whole grains, lean meats, and dairy products with low fat [27].

Choose nutrient-dense foods that are low in calories yet high in vitamins and minerals. Consume less sodium to aid with blood pressure management [11]. Steer clear of adding excess salt to meals and use caution when consuming packaged and processed foods, as they frequently have high sodium content. Drink a lot of water throughout the day to stay well hydrated. Drinking enough water promotes general health and helps keep blood pressure at ideal levels [16]. Preeclampsia incidence was shown to be 20% lower in women who engaged in regular physical exercise, according to a Norwegian study. Pregnant women who engaged in high levels of physical activity in the year before becoming pregnant were 78% less likely to develop preeclampsia than those who engaged in minimal or no exercise during the same time frame, per a New York study. Exercise has been shown by Kasawara et al. to be protective against preeclampsia [28]. Along with this, weight management, stress channeling, regular checkups, and limiting certain substances from dietary habits like alcohol, tobacco, and caffeine [29].

PIH is treated pharmacologically with the goals of regulating blood pressure, averting problems, and guaranteeing the safety of the fetus and mother. The degree of hypertension, the drug's effectiveness, and its safety profile during pregnancy all play a role in the selection of an antihypertensive treatment. Methyldopa alpha-2 adrenergic agonist lowers sympathetic outflow, which lowers blood pressure and causes vasodilation. When coupled with beta- and alpha-blockers, labetalol lowers peripheral vascular resistance without appreciably lowering heart rate or cardiac output [30].

Compared to methyldopa, labetalol lowers blood pressure more quickly and efficiently at both the systolic and diastolic levels. Methyldopa and labetalol have comparable side effects and safety profiles [12]. A study was done to uncover the effectiveness of certain medications in PIH. Blood pressure control, which is defined as a systolic blood pressure of 120-150 mm Hg and a diastolic blood pressure of 70-100 mm Hg, was achieved within six hours with no unfavorable consequences. These unfavorable outcomes included eclampsia, cesarean section for fetal distress up to two hours after the study period ended, severe headaches that required stopping the medication, and hypotension (systolic blood pressure of <120 mm Hg, diastolic blood pressure of <70 mm Hg, or both). The need for more medicine or a modification in the treatment regimen, placental abruption, and adverse effects in mothers linked to worsening pre-eclampsia were the secondary outcomes [31]. Pregnancy-related hypertensive problems can develop after delivery. Preeclampsia or eclampsia postpartum was reported to have developed in 5.7% of the 151 women in one study of the 22 patients who presented to the emergency room with preeclampsia up to four weeks after delivery, 55% were de novo. The causes of postpartum hypertension are complex; fluids and NSAIDs given as part of supportive care may cause blood pressure to rise even more as the body tries to revert to physiology prior to pregnancy, which involves mobilizing extracellular fluid into the intracellular compartment [32,33]. In cases of PIH, fetal monitoring is essential for ensuring the health of the developing baby and for early detection of any problems or signals of fetal distress. Preeclampsia and gestational hypertension are two examples of PIH disorders that can impact fetal growth and placental function, requiring strict monitoring. Fetal growth parameters, such as head circumference, belly circumference, and femur length, are measured by routine ultrasound exams.

In PIH, growth limitation (IUGR) is a worry. Serial ultrasounds can be used to monitor the trends in fetal growth over time. The amount of amniotic fluid surrounding the fetus can be measured via ultrasound. Low amniotic fluid, or oligohydramnios, may be a sign of placental insufficiency. Doppler ultrasonography measures blood flow in the uterine, middle cerebral, and umbilical arteries. Pregnancy-induced hypertension was statistically significantly correlated with a positive family history of the condition; asthma, kidney problems during pregnancy, and gestational age are some of the warning signs to look out for PIH [34].

Discussion

Also known as pregnancy-induced hypertension (PIH), it is a medical disorder characterized by increased blood pressure during pregnancy. It frequently occurs if it is left untreated in pregnancies that are 20 weeks or longer. It can lead to significant complications for the woman along with the unborn baby. Ninety-nine of the pregnancy complications are associated with the placenta; therefore, insufficient blood flow hypes up blood pressure. Some of these risk factors include a history of PIH or hypertension in previous pregnancies, younger age, non-white ethnicity, high body mass index, and multiple pregnancies. Observably, women with existing diseases such as diabetes, renal disease, or hypertension are fundamentally more susceptible [9]. Some of these risk factors include poor diet, lack of exercise, and the condition known as obesity, which can cause Parkinson’s illness. Indicative of a severe form of hypertension distinguished by features of the target organ-damaging process typical of a renal-hepatic form of hypertension. Other complications, such as preeclampsia, which in some cases can be lethal along with this seizure. A study shows that lifelong use of antihypertensive medication to treat any type of pregnancy-associated hypertension reduces the rate of developing severe hypertension by 50%. This may be acceptable to some, if not most, to make a change in practice towards more severe therapy. This might be particularly important in settings that have poor density and limited capacity or insufficient human resources for managing hypertensive emergencies and urgent conditions [6].

PIH is an onset of hypertension that occurs after 20 weeks of pregnancy, without a previous history of it, and is considered a serious public health problem. The exact cause of PIH is still not established; however, if it reaches conditions such as preeclampsia and eclampsia, it poses a great danger for both the mother and the fetus. The nature of predisposing factors, implications, and risk factors are complex in PIH; therefore, it is crucial to appraise its multi-factorial presentation, complications, and management to prevent adverse effects on the mother and fetus [34]. PIH can manifest clinically in several ways. Although some of the women may be asymptomatic, some may be symptomatic, and this includes hypertension, which is described as systolic blood pressure of 140 mmHg or above or diastolic of 90 mmHg or above as confirmed by two consecutive readings taken at least four hours apart. Proteinuria occurs together with the symptoms in some patients and may include headache, vision problems, and epigastric discomfort. As to the extrapolation of PIH implications, it also stressed the importance of prenatal visits and paying extra attention to blood pressure readings during the pregnancy period [35].

The implications of this are dramatic in view of the many facets of life that are touched in the case of PIH. It also increases the risk of preeclampsia, a related condition in which the woman also has both high blood pressure and protein in her urine. Preeclampsia can progress to eclampsia, which is characterized by convulsions or coma and is fatal. On the complications, placental abruption, disseminated intravascular coagulation, and even maternal mortality may result from the same. About the fetus, PIH entails chances of intrauterine growth restriction (IUGR), whereby the baby does not grow adequately in the womb due to poor blood flow to the placenta. This results in low birth weight, and since low-birth-weight infants are born with feeble respiratory health and great susceptibility to infections and developmental complications, this could lead to complications in the newborn. On the same note, PIH may increase the possibility of preterm birth and hence compromise the lives of the fetuses. Though the exact causes of PIH are still unclear to the researcher, the following are known risk factors. Others are diseases that a person develops before the incident, such as hypertension, diabetes, or kidney conditions. Further, age factors that include ancestry with PIH, obesity, and greater than 40 years of age are considered risk factors. Knowledge of these risks makes it possible to practice differential detection and also develop prevention measures [36].

The foundation of PIH management is the identification of the condition during the earlier stages, as well as constant supervision. Appointments with the doctor and blood pressure and urine examinations at least once a week are recommended. Care for the infant with PIH also depends on the stage of pregnancy and degree of the disease; an individual plan of treatment is drawn up. At early stages, depending on the symptoms, the patient may be placed under close supervision and recommended to change their diet and get more rest, among others. However, if the condition is severe, then certain medications would be required to regulate blood pressure as well as the status of the fetus. When PIH has escalated to preeclampsia or eclampsia, the management becomes more intensive. Close observation under the doctor's care and possible modification of their dosage is required, and the individual has to be hospitalized. Depending on the severity and gestational age, then it would be advised to have the baby delivered to protect both the life of the mother and the unborn child. Although extensive studies have been done on the aspects of PIH management and increased knowledge of the conditions, research is still essential in the future. Decoding the risk factors for PIH allows for better strategies of primary prevention to be designed in the future. Moreover, improvements in diagnostics and any further research in other forms of treatment continue to be investigated. Studies on other supposed biomarkers that may be useful in the early identification of PIH or the extent of the disease go a long way in preventing or managing it [37,38].

## Conclusions

A multifaceted strategy is needed to address pregnancy-induced hypertension, including a thorough understanding of its intricate pathogenesis, the application of potent pharmaceutical and lifestyle treatments, and vigilant fetal monitoring. Regular checkups are important to diagnose any anomaly, if present, which gives extra leeway for healthcare professionals. Immediate identification and prompt treatment are essential for lowering the chances of unfavorable consequences for mother and child. To further improve the care and prognosis for women with PIH, further research, and better clinical procedures are required. If the expected mother has a genetic predisposition for hypertension, then extra care needs to be taken to ensure an ideal positive outcome. More specifically, teaching these expectant mothers about the signals and signs of PIH in an effort to ensure early reporting and intervention. Developing and implementing the operational guidelines for handling severe PIH occurrences that may call for immediate response accompanied by shipment if appropriate.
